# AGE DIFFERENCES IN PRIOR EXPERIENCES USED TO COPE WITH COVID-19 STRESSORS

**DOI:** 10.1093/geroni/igad104.2686

**Published:** 2023-12-21

**Authors:** Maria Kurth, Heidi Igarashi, Carolyn Aldwin

**Affiliations:** Penn State University, University Park, Pennsylvania, United States; Oregon State University, Corvallis, Oregon, United States; Oregon State University, Corvallis, Oregon, United States

## Abstract

Despite increased physiological vulnerability to COVID-19 (CDC, 2022), older adults reported fewer mental health consequences than younger adults (APA, 2022). The ability to draw upon prior experiences may be a source for enhanced well-being in late life (Aldwin & Igarashi, 2016; Charles, 2010). Older adults reported various prior experiences and resources for coping with the COVID-19 pandemic (Galcia et al., 2021; Herron et al., 2021), but age differences were not examined. We collected online surveys from adults 50+ (N = 235; Mage = 71.35, SD = 7.39; 74% female; 92% White) between 04/28–05/04/2020. A directed content analysis guided application of pre-existing (Aldwin et al., 1996b) and novel codes to the 60% who responded to an open-ended question of which prior experiences informed coping with COVID-19 pandemic problems. The final coding scheme had 13 codes (Kurth et al., 2022; Krippdendrof ∝ = .82) grouped into three categories: similar experiences, adversity across the lifespan, and personal resources. No age differences emerged in likelihood of providing any of these responses between the midlife (51 – 64; n = 35), young-old (65 – 74; n = 141), and old-old groups (75+; n = 57). Further, there were no age differences in providing a response that fell into the similar experience, adversity across the lifespan, or personal resource categories, supporting the age and well-being hypothesis. Findings suggest that even when exposed to a chronic and uncontrollable stressor, older adults leveraged their advantage of past experiences and accumulated resources to inform coping strategies.

